# Genetic Diversity of *Sitobion avenae* (Homoptera: Aphididae) Populations from Different Geographic Regions in China

**DOI:** 10.1371/journal.pone.0109349

**Published:** 2014-10-30

**Authors:** Juan-Juan Xin, Qing-Li Shang, Nicolas Desneux, Xi-Wu Gao

**Affiliations:** 1 Department of Entomology, China Agricultural University, Beijing, PR China; 2 College of Plant Science and Technology, Jilin University, Changchun, PR China; 3 French National Institute for Agricultural Research (INRA), Paris, France; University College Dublin, Ireland

## Abstract

*Sitobion avenae* is a major agricultural pest of wheat in China. Using microsatellite markers, we studied the potential gene flow, genetic diversity, genetic differentiation, and genetic structure of seven *S. avenae* populations from different regions of China (Beijing, Hebei, Henan, Hubei, Jiangsu, Shandong, and Shanxi provinces). The populations from Henan, Shandong, and Jiangsu showed high levels of genic and genotypic diversity. By contrast, the genic diversity in the Beijing and Hebei populations was much lower. Despite this low genic diversity, the genotypic diversity of the Beijing population was higher than that of all of the other populations, except those from Jiangsu and Shandong. Overall, the genetic divergence among the seven *S. avenae* populations tested was high, though there was almost no differentiation between the Shandong and Henan populations. We observed significant negative correlation between the strength of gene flow and the geographic distances among populations. Based on genetic analysis, the seven *S. avenae* populations studied can be divided into four distinct clusters; (i) Hubei, (ii) Shanxi, (iii) Beijing and Hebei, and (iv) Shandong, Henan, and Jiangsu. The present results provide a basis for potentially optimizing integrated pest management (IPM) programs in China, through adapting control methods that target biological traits shared by various populations of the same genotype.

## Introduction

Several wheat aphid species are major agricultural pests in China, notably *Sitobion avenae, Rhopalosiphum padi* (L.), *Schizaphis graminum* (Rondani), and *Acyrthosiphon dirhodum* Walker [1]. Of these, *S. avenae* is the most dominant and destructive wheat aphid in China [2], [3]; it affects about 13 million hm^2^ per year, and yield losses can be up to 40% [4]. Wheat aphids infest cereal crops and cause direct economic losses through sucking sap, and indirect losses by vectoring plant viruses [5].

Wheat aphids have complex life cycles that are known to be highly affected by climate [6]. Some aphid species exhibit great flexibility in the selection of their reproductive mode [7]–[9]. All lifecycle types reproduce parthenogenetically for most of the year; some species have an annual sexual phase [10]. The lifecycle of cyclic parthenogenic species have a sexual reproduction phase once a year, and overwinter as asexually produced eggs; this is a more reliable strategy when winters are harsh [11]–[16]. Cyclic parthenogenesis is the dominant mode of reproduction, especially in the regions with harsh winters [15], [17]. However, if the climatic conditions allow, some clones are obligate parthenogenic, they do not respond to autumnal cues and have no sexual phase. There are also intermediate types which employ both sexual and parthenogenetic reproduction [6], [9], [15], [18]. A study showed that the asexual genotypes of *Rhopalosiphum padi* had higher genetic variation in fitness compared to the sexual genotypes [19], indicating that the reproductive mode greatly impacts the fitness of wheat aphids.

*S. avenae* can survive on numerous plant species, including all of the cereals, many other monocots, and certain dicots [20]. Divergent selection on different host plants greatly influences the diversification of the aphids, and imposes considerable selective pressure on aphids' evolution [21]. Host-transfer experiments for clones of *S. avenae* collected from oat and barley showed that their fitness traits differed significantly, indicating a genetic basis for their differentiation [22].

Aphids have complex lifecycles and adaption to diverse hosts; this means that any given geographical population of aphids will not be homogeneous [23]. If the migration scale is large enough, the population genetic structure and evolutionary trajectory will be influenced [24]. As different kinds of aphids have different abilities to fly and face different pressures leading to migration, they act out different migration behaviors [25]. The flight ability of winged aphids is very weak [26], [27], and they migrate for long distances by largely depending on wind forces [28].

Clonal selection is very important in the study of population genetic structures of wheat aphids. Ecological adaption may occur quickly because of the rapid propagation of asexual offspring [29]. There are many factors influencing clonal selection. These include climatic, host plant, microclimates, crop density, natural enemy pressure, and resistance to pesticides [15], [30], [31].

It is difficult to understand the ecology of wheat aphids, as they tend to migrate with the wind [32]. Additionally, wheat aphids are small and have short life spans and large population sizes. Populations can be diluted rapidly in the air and migrate frequently [26]. Therefore, it is difficult to monitor the migration of wheat aphids using ecological approaches. Currently, polymorphic genetic markers have been widely used in the studies of population ecology [25].

In the past 20 years, many studies addressing the flying behavior and genetic structure of aphids populations have been conducted using microsatellite markers [15], [26], [27], [33], [34]. As noted, wheat aphids have different lifecycles based on the climate conditions of various geographic regions; the most suitable clone lineages for a given area are selected. Due to lifecycle strategy and clonal selection, studies of the aphids in the same region, but in different years, may also present different population genetic structures [15], [35]. However, the gene flow among different geographical populations can also be reflected in population genetic differentiation and population structure [34]–[36]. The management methods used to control wheat aphids can lead to new phenotypes emerging to overcome strong pressures such as pesticide applications, so quantification of the genetic diversity of populations is very important for the management of wheat aphids [37].

In the present study, we used five microsatellite loci (Sm10, Sm11, Sm12, Sm17, and Sa∑4), which had been used previously [15], [25], [38], [39], [40], to characterize the potential gene flow, genetic diversity, genetic differentiation, and genetic structure of seven *S. avenae* populations from seven different geographic regions in China. To interpret the population structure and dynamics of *S. avenae*, we need to better understand the importance of migration and clonal selection, which may have relevance in forecasting aphid outbreaks. Our results also provide a foundation for optimizing integrated pest management (IPM) programs through adapting control methods according to biological traits shared by various populations of the same genotype.

## Materials and Methods

### Aphid Sample Collection

**Figure 1:**
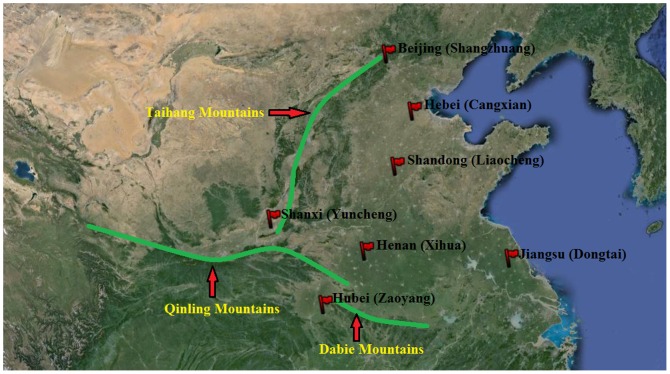
*S. avenae* sampling sites in China.

Wingless adults of *S. avenae* were collected from seven sites across six provinces (Hebei, Shandong, Henan, Shanxi, Jiangsu, and Hubei province) and the Beijing municipality of China. These are the main wheat producing provinces of China. The site locations are presented in Figure 1. The fields, where the *S. avenae* collected from, are agricultural experiment fields belong to plant protection research stations at Cangxian (Hebei province, China), Liaocheng (Shandong province, China), Xihua (Henan province, China), the Yanhu distriction of Yuncheng (Shanxi province, China), Dongtai (Jiangsu province, China), Zaoyang (Hubei province, China) and the Agricultural Experiment Station of China Agricultural University (Beijing, China), respectively. 30∼40 individuals were collected from each site, and each aphid was taken from a different field in order to minimize resampling of the same clone. Samples that were collected from the same region were considered as one ‘population’. The collected specimens were taken back to the laboratory in 100% ethanol.

### DNA Extraction and PCR of Microsatellite Loci

Genomic DNA was extracted from wingless adult aphid individuals using DNAVzol (Vigorous Biotechnology Beijing Co., Ltd.), according to the manufacturer's instructions.

Two hundred forty six aphids were examined at five microsatellite loci (Sm10, Sm11, Sm12, Sm17, and Sa∑4). The microsatellite loci of Sm10, and Sm11, Sm12, Sm17 were isolated from *S. miscanthi* [38], and Sa∑4 was isolated from *S. avenae*. Sm11 is x-linked, and the other three loci are autosomal [40]. The SaΣ4 locus is also autosomal [15]. The primer sequences of 4 microsatellite loci (Sm10: 5′-TCTGCTGCATTACTGTTGGC-3′, 5′ -TCGTCTACTTCGCCGTCA-3′; Sm11: 5′-TCTGCTGCATTACTGTTGGC-3′, 5′ -TCGTCTACTTCGCCGTCA-3′; Sm17: 5′-TCTGCTGCATTACTGTTGGC-3′, 5′ -TCGTCTACTTCGCCGTCA-3′; Sa∑4: 5′-TCTGCTGCATTACTGTTGGC-3′, 5′ -TCGTCTACTTCGCCGTCA-3′) were reported by Simon *et al.* (1999) [15]. The primer sequences of locus Sm12 (5′-CACCATCGCGTTTCATCTTA-3′; 5′-ACTCCCAACCTCTGATGAGC-3′) were reported by Llewellyn *et al.* (2003) [25]. They have verified before application.

The PCR conditions were the same as those of Simon *et al.* (1999) [15]. PCR reactions were performed in a 20 µL reaction volume. Each reaction mixture contained 0.2 µL of 5 U/µL rTaq polymerase, 2.0 µL of each 2.5 umol dNTP and 2.0 µL of ×10 buffer (TaKaRa Biotechnology (Dalian) Co.,Ltd.), 1 µL of 10 µM of each primer (SANGON), and 1 µL of DNA template (approximately 10 ng). The PCR products were examined using an ABI3730*1 instrument and the allele sizes were analyzed using Genemapper 3.0 software (Applied Biosystems).

### Data Analysis

The genotype data is presented in File S1. Departure from Hardy-Weinberg equilibrium (HWE) was tested under the hypothesis of heterogeneity deficit and excess using Genepop [41], [42]. The score test (U test) was used. Linkage disequilibrium among the microsatellite loci were also tested using Genepop [41], [42]. The null hypothesis was “genotypes at one loci are independent from genotypes at the other loci”, and the default test statistic was the log likelihood ratio statistic (G-test). During the processes of identification and isolation of microsatellite sequences using primers and amplification by PCR, the following errors can occur: 1. One or more alleles fail to amplify during PCR (null alleles); 2. Slight changes occur in the allele sizes during PCR (stuttering); 3. Large alleles do not amplify as efficiently as small alleles. We detected these errors using Micro-Checker v2.2.3 [43]. This application calculates the frequency of any null alleles detected, using the methods described by Chakraborty *et al*. (1992) [44] and Brookfield (1996) [45]. The indices of genetic diversity including the number of alleles (*N_a_*), the richness of alleles (*A_r_*), the observed heterozygosity (*H_O_*), the expected heterozygosity (*H_E_*), gene diversity (*H_S_*), and the inbreeding index (*F_IS_*) for each population were calculated using FSTAT v2.9.3 [46]. *N*a is the mean number of alleles in each sample. It can be calculated according to the following equation:
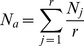



*N_j_* is the number of alleles at microsatellite loci *j* in one sample. *r* is the number of all loci. The richness of alleles (*A_r_*) is the mean of *R_s_* (allelic richness per locus and population) across all microsatellite loci. *R_s_* is a measure of the number of alleles and is independent of sample size, hence allowing one to compare *R_s_* between different sample sizes. However, the observed number of alleles in a sample is highly dependent on sample size. To bypass this problem, El Mousadik and Petit (1996) [47] suggested the adaption of the rarefaction index of Hurlbert (1971) [48] to population genetics. The principle is to estimate the expected number of alleles in a sub-sample of 2*n* genes, given that 2*N* genes have been sampled (*N*≥*n*). In FSTAT, *n* is fixed as the smallest number of individuals typed for a locus in a sample. Allelic Richness is then calculated according to the following equation:
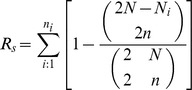



Where *N_i_* is the number of alleles of type *i* among the 2*N* genes. Note that each term under the sum corresponds to the probability of sampling allele *i* at least once in a sample of size *2n*. In FSTAT, estimates of gene diversity per locus and sample use an unbiased estimator [49]: 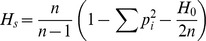



Where *n* is the size of a sample. *p_i_* is the frequency of allele A_i_ in a sample. *H_o_* is the observed proportion of heterozygotes in a sample. The multilocus genotypes (MLG) were classified based on the length of alleles, using software that we developed in house. The genotype diversity (*K*) of each population was calculated as follows: *K = G/N*, where *G* is equal to the number of MLGs, and *N* is the number of samples [25].

*F*-statistics (*F_IT_*, *F_IS_*, and *F_ST_*) and pairwise *F_ST_* [50], were estimated using FASTAT v2.9.3 [46]. Nei's standard genetic distance (*D_S_*) [51] and Nei's genetic distance (*D_A_*) [52] were calculated using the Dispan program [53]. An indirect estimate of gene flow was calculated as 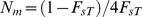 [54]. To analyze the relationship between *F_ST_* and geographic distance, a Mantel test was carried out. The matrices of pairwise *F_ST_* and the geographic distance between two populations was transformed by Genepop (the geographic distances were transformed to the natural logarithm (*Ln*) of geographic distances in kilometers), and a regression was performed using SPSS 17.0.

To identify the population structure, two clustering methods were used. First, we constructed phylogenetic trees (dendrograms) using the unweighted pair group-method with arithmetic mean (UPGMA) [55], based on Nei's genetic distance (*D_A_*) using the Dispan program. Second, factorial correspondence analysis (FCA) was implemented in Genetix v4.04 [56], to examine the three-dimensional spatial distribution of genetic variation for each individual.

An analysis of molecular variance (AMOVA) was implemented in Arlequin v3.0 [57], to confirm population clusters and to examine the component variance of genetic differentiation among populations.

## Results

### Genetic Diversity

We screened 246 aphids collected from six provinces and the Bejing municipality of China for five microsatellite loci. There was no large allele dropout or stuttering observed in the result. Of the 35 tests, there were 6 tests in which null alleles were found to exist; they appeared at the Sm11 and Sm17 microsatellite loci. However, no particular microsatellite loci had null alleles in all of the populations. The frequencies of the null alleles were not more than 0.08 (Table 1). In the 70 linkage disequilibrium tests, there were 12 tests with significant linkage (Table 1). However, no fixed combination of any two microsatellite loci showed significant linkage in all of the populations, and the 5 microsatellite loci selected here can estimate genetic mutation independently. Five populations deviated from HWE significantly because of heterozygote deficit; both Beijing and Shanxi populations were the exception to this. The Shanxi population deviated from HWE because of heterozygote excess (Table 1). The *F_IS_* values of populations were positive, except for the Beijing and Shanxi populations. The *F_IS_* of the Shanxi population was the most negative (Table 2).

**Table 1:** Outcomes of null allele frequency, linkage disequilibrium test, and HWE test.

Populations	Beijing	Hubei	Hebei	Henan	Jiangsu	Shandong	Shanxi
Null allele frequency (*r*)							
Sm10	—	—	—	—	—	—	—
Sm11	—	—	—	0.06	—	0.07	—
Sm12	—	—	—	—	—	—	—
Sm17	0.08	—	0.03	0.06	0.08	—	—
Sa∑4	—	—	—	—	—	—	—
Linkage disequilibrium test (*p*-value)							
Sm10-Sm11	0.51	0.01*	0.03*	0.75	0.34	0.64	0.04*
Sm10-Sm12	0.09	0.07	0.00*	1.00	0.74	1.00	0.12
Sm11-Sm12	1.00	0.00*	0.01*	0.19	NA	1.00	1.00
Sm11-Sm17	NA	0.01*	0.25	0.00*	0.94	0.00*	1.00
Sm12-Sa∑4	0.46	0.06	NA	NA	0.03*	NA	1.00
Sm10-Sm17	0.28	0.02*	0.21	0.04	0.11	0.77	0.06
Sm10-Sa∑4	0.24	0.76	NA	NA	0.66	0.61	0.97
Sm11-Sa∑4	1.00	0.26	NA	NA	1.00	0.46	1.00
Sm12-Sm17	0.21	0.02*	0.22	0.13	0.05	1.00	0.62
Sm17-Sa∑4	NA	0.39	NA	NA	0.60	0.74	0.17
HWE test (*p*-value)							
heterozygote deficit	0.58	0.00^a^	0.01^a^	0.00^a^	0.00^a^	0.00^a^	1.00
heterozygote excess	0.42	1.00	0.99	1.00	1.00	1.00	0.00^a^

“—”: There were no null alleles detected.

“*”: p<0.05, significant linkage; “NA”: no contingency table.

“a” : P<0.05, significant deviation from Hardy-Weinberg equilibrium.

**Table 2:** Indices of genetic diversity for the seven *S. avenae* populations of China sampled in 2012.

	Beijing	Hubei	Hebei	Henan	Jiangsu	Shandong	Shanxi
*N*	40	35	36	32	36	31	36
*H_S_*	0.390	0.530	0.487	0.741	0.637	0.728	0.546
*N_a_*	6.0	7.6	6.8	15.2	13.0	13.0	7.2
*A_r_*	3.65	4.35	3.69	8.36	6.11	7.15	4.10
*F_IS_*	−0.075	0.027	0.036	0.079	0.003	0.122	−0.413
*H_O_*	0.42	0.52	0.59	0.85	0.64	0.65	0.77
*H_E_*	0.39	0.52	0.60	0.92	0.62	0.72	0.54
MLG	37	31	25	29	36	31	26
*K*	0.925	0.886	0.694	0.906	1.000	1.000	0.722
within	3	4	4	3	0	0	6
among	0	0	0	0	1	1	0

*H_O_*, observed heterozygosity; *H_E_*, expected heterozygosity; *H_S_*, gene diversity; *N_a_*, mean numbers of alleles; *Ar*, allelic richness based on eight samples per population; MLGs, number of multilocus genotypes; #within, number of MLGs shared within a population; #among, number of MLGs shared among populations; *K*, index of global genotypic diversity (MLGs /*N*); *F_IS_*, the inbreeding index. The seven populations were named by their sampling locations, Beijing, Hubei, Hebei, Henan, Jiangsu, Shandong, and Shanxi, respectively.

Our results suggested that *S. avenae* in China have a high level of genetic diversity. The indices of genetic diversity of the seven populations are listed in Table 2. The results show that the populations of Henan, Shandong, and Jiangsu have relatively high levels of genic and genotypic diversity. The values of *N_a_*, *Ar*, and *H_S_* present in the Henan population (15.2, 8.36, and 0.74, respectively) were higher than the values for the other populations. The genic diversity level of the Beijing population was the lowest; the values of *Na*, *Ar*, and *H_S_* of the Beijing population were 6, 3.65, and 0.39, respectively. However, the genotypic diversity of Beijing population was higher (*K* = 0.925) than all of the other populations, except for the Jiangsu and Shandong populations.

We observed 215 multilocus genotypes from the 246 aphids tested. The same genotypes were observed within the Hubei, Henan, Shanxi, Hebei, and Beijing populations, but the same genotype was not found within the Jiangsu and Shandong populations. However, one individual in 31 aphids from Shandong was found to share the same genotype with one individual from Jiangsu province. There were not any genotypes shared among the other five populations.

### Genetic Differentiation

Population differentiation was analyzed using pairwise *F_ST_* values (Table 3). The pairwise *F_ST_* values between the Beijing and the other populations, except for Hubei, were generally high (0.2589∼0.4186); high levels of differentiation were also detected between Hubei and the other populations (0.2137∼0.3626). The pairwise *F_ST_* value between the Shandong and Jiangsu populations (*F_ST_* = 0.1012) was close to the value between the Beijing and Hebei populations (*F_ST_* = 0.1055), which were lower than the values for the other populations, with the exception of the value between the Henan and Shandong populations (*F*_ST_ = 0.0383).

**Table 3:** Pairwise *F_ST_* value among the seven *S. avenae* populations of China.

	Beijing	Hubei	Hebei	Henan	Jiangsu	Shandong
Hubei	0.4186					
Hebei	0.1055	0.3626				
Henan	0.2604	0.2137	0.2015			
Jiangsu	0.3547	0.3182	0.2852	0.1611		
Shandong	0.2589	0.2253	0.1911	0.0383	0.1012	
Shanxi	0.3836	0.3320	0.2648	0.1654	0.2724	0.1858

*F_ST_*≤0.05, indicates little differentiation among populations; 0.05<*F_ST_*≤0.15, middling differentiation; (iii). 0.15<*F_ST_*≤0.25, high differentiation; *F_ST_*>0.25, significant divergence. The seven *S. avenae* populations were named by their sampled locations, Beijing, Hubei, Hebei, Henan, Jiangsu, Shandong, and Shanxi, respectively.

According to an analysis of molecular variance (AMOVA), the *S. avenae* populations sampled had high levels of genetic differentiation (*F_ST_* = 0.2547) (Table 4). Genetic variation between populations accounted for 25.47% of the total genetic variation.

**Table 4:** The composition of variation (average over 5 loci).

Source of variation	Sum of squares	Variance components (V_i_)	Percentage variation	Fixation Indices
Among population	176.279	0.49323Va	25.47	*F_ST_* = 0.2547
Among individuals within populations	272.715	−0.09396Vb	−4.85	*F_IS_* = −0.0651
Within individuals	319.500	1.53724Vc	79.38	*F_IT_* = 0.2062
Total	768.494	1.93651		

Va, the variance among populations; Vb, the variance whin population; Vc: the variance within individuals. *F_ST_*, the average of genetic differentiation among populations; *F_IS_*, the average of inbreeding coefficient among individuals within populations; *F_IT_*, the average of total inbreeding coefficient.

The migration number among different geographical populations has been proposed to be represented as gene flow (*N_m_*) [54]. There is little divergence if *N_m_*>1. However, if *N_m_*<1, it means that gene flow could not counteract the divergence caused by genetic drift [58]. In the present study, the level of genetic differentiation among the seven *S. avenae* populations was *N_m_* = 0.899 (Table 5), and the gene flow was not large enough to offset the genetic drift. The biggest gene flow (*N_m_* = 6.277) was observed between the Henan and Shandong populations (Table 6). The values of *N_m_* between Beijing and the other populations were less than 1, except for the Hebei population. The gene flow between the Hubei population and the other populations was also low (*N_m_*<1).

**Table 5:** Summary of the *F*-statistics at five microsatellite loci.

	*F_IT_*	*F_IS_*	*F_ST_*	*N_m_*
Sm10	0.012	−0.086	0.090	2.528
Sm11	0.502	0.082	0.457	0.297
Sm12	0.033	−0.183	0.182	1.124
Sm17	0.282	0.021	0.267	0.686
Sa∑4	0.126	0.038	0.092	2.467
Mean	0.191	−0.026	0.218	0.899

*F_IT_*: the average of total inbreeding coefficient; *F_IS_*: the average of inbreeding coefficient among populations; *F_ST_*: the average of genetic differentiation; *N_m_*: gene flow, 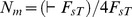.

**Table 6:** The number of individuals that migrated among differential regions.

N_m_	Beijing	Hubei	Hebei	Henan	Jiangsu	Shandong
Hubei	0.3472					
Hebei	2.1197	0.4395				
Henan	0.7101	0.9199	0.9907			
Jiangsu	0.4548	0.5357	0.6266	1.3018		
Shandong	0.7156	0.8596	1.0582	6.2774	2.2204	
Shanxi	0.4017	0.5030	0.6941	1.2615	0.6678	1.0955

*N_m_*, gene flow, 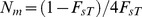.

Overall, the genetic divergence of the populations had significant positive correlation (*r^2^* = 0.401, *p* = 0.002) with geographic distance, according to the results of the Mantel test.

### Population Structure

We used two methods for clustering analysis, and a small divergence appeared between the two sets of results.

**Figure 2:**
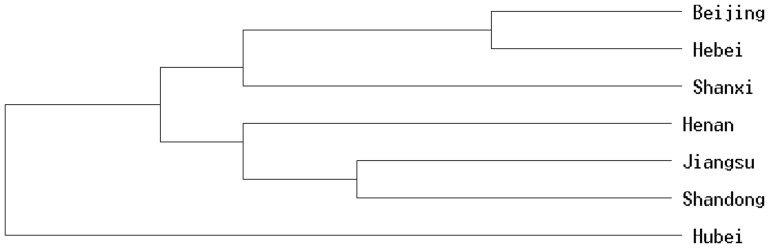
UPMGA Phylogenetic tree of seven different *S. avenae* geographic populations in China.

First, we classified the seven geographical populations into three clusters using the UPGMA method (Figure 2): (i) Hubei, (ii) Shanxi, Beijing and Hebei, and (iii) Shandong, Henan, and Jiangsu.

**Figure 3:**
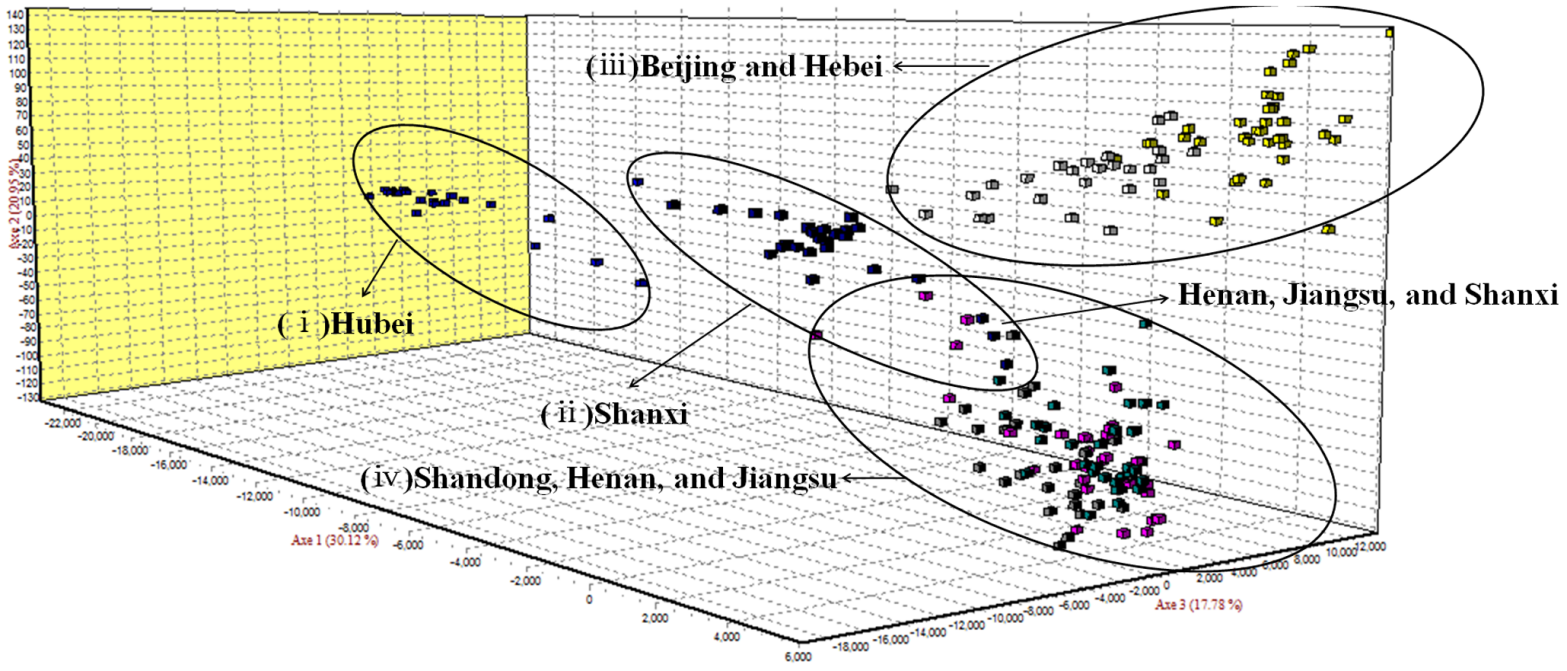
Three-dimensional factorial correspondence analysis (FCA) of *S. avenae* sampled in 2012. The circles indicate populations that cluster according to geography.

Second, the FCA results divided the seven populations into four groups (Figure 3); (i) Hubei, (ii) Shanxi, (iii) Beijing and Hebei, and (iv) Shandong, Henan, and Jiangsu. The only differentiation between results of the two clustering methods was that the Shanxi population formed its own cluster in the results of the FCA method.

However, the two kinds of genetic distance (*D_S_* and *D_A_*) (Table 7) between the Shanxi and Beijing populations were larger than between Shanxi and the other populations, except for Hubei. Therefore, the Shanxi and Beijing populations cannot be classified into a single group.

**Table 7:** The standard genetic distance (*D_S_*) and Nei's unbiased genetic distance (*D_A_*) of pairwise comparisons among the seven populations.

	Beijing	Hubei	Hebei	Henan	Jiangsu	Shandong	Shanxi
Beijing	—	1.0144	0.0890	0.5421	0.9047	0.5294	0.8219
Hubei	0.7344	—	0.8859	0.5784	1.0331	0.6196	0.8590
Hebei	0.1914	0.6757	—	0.4638	0.7195	0.4123	0.4870
Henan	0.5674	0.7286	0.5835	—	0.5557	0.1233	0.3740
Jiangsu	0.5864	0.7111	0.5447	0.5470	—	0.2811	0.7536
Shandong	0.5050	0.7147	0.4976	0.3532	0.3294	—	0.4354
Shanxi	0.5382	0.6810	0.3746	0.5354	0.5196	0.5318	—

“upper triangle”, Nei's standard genetic distance (*D_S_*); “lower triangle”: Nei's genetic distance (*D_A_*). The seven populations were named by their sampled locations, Beijing, Hubei, Hebei, Henan, Jiangsu, Shandong, and Shanxi, respectively.

We classified the seven populations into 4 groups (i. Hubei; ii. Shanxi; iii. Beijing and Hebei; iv. Shandong, Henan, and Jiangsu.), and conducted AMOVA to detect the variance components. The results showed that the variances among groups and among populations within groups accounted for 18.34% and 9.53% of the total variance, respectively (Table 8), and that the variance between groups was significantly larger than the variance among populations within a particular group (*F_CT_* = 0.18399). This result indicates that gene flow among the four groups was strictly restricted. The AMOVA results thus confirmed the conclusions of the clustering analysis.

**Table 8:** Analysis of molecular variances (AMOVA) of microsatellites in the seven populations.

Source of variation	Sum of squares	Variance components	Percentage variation	Fixation Indices
Among groups	142.425	0.36698	18.33888	*F_CT_* = 0.18399
Among populations within groups	33.855	0.19082	9.53602	*F_SC_* = 0.11678
Among individuals within population	272.715	−0.09396	−4.69544	*F_IS_* = −0.06510
Within individuals	319.5	1.53724	76.82053	*F_IT_* = 0.23179
Total	768.494	2.00108		

V_a_, variance components among groups; V_b_, variance components among populations but within groups; V_c_, variance components among individuals within population; V_d_, variance components within individuals; *F_CT_*, *F*_SC_, *F_IS_*, and *F_IT_* are inbreeding coefficients.

## Discussion

*S. avenae* is a migratory pest insect that is widely distributed throughout the wheat growing regions of China [59]. In the present study, we analyzed the population genetic diversity, population genetic differentiation, and genetic structure of seven different *S. avenae* geographical populations in China using microsatellite marker technology. These research results provide a basis for efforts to optimize integrated pest management (IPM) programs through adapting control methods which target the biological traits shared by various populations from the same genotype.

### High Level of Genetic Diversity

The genetic diversity of aphids is influenced by many factors such as climate, host plants, topography, and physiognomy [60]. Wheat aphid populations frequently exhibit high levels of genetic variation [61]. In the present study, a high level of genetic diversity was detected at all of the five microsatellite loci. There were 32, 41, 35, 60, and 13 alleles at the Sm10, Sm11, Sm12, Sm17, and Sa∑4 loci, respectively. The genetic diversity level in our study (average  = 36.2 alleles/locus) was much higher than that detected by Guo *et al.* (2005) (average  = 9.6 alleles/locus) [61]. In particular, the genic diversity of the Henan and Shandong populations was higher than the diversity of the other populations. This may be due to the fact that their geographic location had more gene flow with the surrounding wheat production regions. In our results, genotypic diversity was also quite high. Two hundred fifteen multilocus genotypes were distinguished in the 246 individual samples. A given genotype was rarely present in the different geographic populations, which may result from a prevalence of sexual reproduction.

Many factors can affect the heterozygosity of populations. The expected heterozygosity (*H_E_*) of the Hebei, Henan, and Shandong populations was lower than their observed heterozygosity (*H_O_*). According to previous aphid genetic studies, factors like inbreeding, the Wahlund effect, and the presence of null alleles and/or clonal selection have been implicated in the generation of heterozygote deficits [39], [62]. As *S. avenae* has the ability for long-range dispersal, and their host plants are common and widespread, strong inbreeding and the Wahlund effect might not occur in this aphid [39], [63]. There are also many reasons for heterozygote excess, including rarely migrating between locations, asexual lineage expansion, and/or the accumulation of heterozygosity by mutation in longer-term parthenogenesis [64]. In our results, the greatest heterozygosity excess was present in the Shanxi population, and the *F_IS_* value of the Shanxi population was extremely negative. This might be associated with the geography of Yuncheng of Shanxi. Shanxi province is mountainous, which might restrict aphid migration.

### Genetic Differentiation and Genetic Structure

The genetic structure of populations reflects the interaction of genetic drift, mutation, gene flow, migration, and natural selection [25]. There is absolutely no genetic differentiation among populations when *F_ST_* = 0, and when the *F_ST_* = 1, the populations can be recognized to have differentiated completely. The threshold of *F_ST_* value can be divided into 4 levels: (i). *F_ST_*≤0.05, indicates little differentiation among populations; (ii). 0.05<*F_ST_*≤0.15, middling differentiation; (iii). 0.15<*F_ST_*≤0.25, high differentiation; (iv). *F_ST_*>0.25, significant divergence [65].

In the present study, there was a high level of genetic differentiation (*F_ST_* = 0.2547) present in the *S. avenae* populations of China. Various factors might affect the divergence of *S. avenae* populations in different sites, including reproductive mode, clonal selection, migration, and so on. Additionally, genetic differentiation might be accelerated by the high level of variation in *S. avenae* population size [66].

A study showed that the aphid clones on the winter host plant did not significantly contribute to the spring/summer population build-up in the cereal fields over short distances, but that the density of sources for early migrants, on a regional scale, is important for the establishment of the population [67]. Therefore, the migration of aphids plays an important role in genetic structure. Aphids can become suspended in the air, and thus fly for long distances with the wind [10], [68]. This flight phenomenon can overcome the combination of the forces of selection, genetic drift, and mutation, if the scale of the migration of *S. avenae* is large enough [25]. The time we collected the wheat aphids samples was the peak time of their breeding. It is known that when the population density of *S. avenae* is high enough and if the host plant cannot supply adequate nutrition, winged aphids will be produced, and a large scale migration can occur. In our results, there was little differentiation between the Henan and Shandong populations (*F_ST_* = 0.0383), which might be attributed to large scale migration and/or the local pest management strategies. The Mantel test showed that the genetic divergence of the populations had a significant positive correlation with the geographic distances between the populations. This means that the gene flow between populations was significantly associated with the distance among the locations, and that the gene exchange among the different geographic populations could be affected by geographical isolation. For example, the genetic divergence between Beijing (the most northern site in our study) and Hubei (the most southern site in our study) populations was the highest as compared to the others.

However, geographical distance didn't explain the study results completely. According to the results published by Huang *et al.* (2013) [69], some vital life-history traits (the developmental time of the instars of nymphs, postreproductive timing, and total lifespan of adults, and so on) were different for aphid populations living North or South of the Qinling Mountains. The divergence and gene flow among *S. avenae* populations might be impacted by the barrier of the Qinling Mountains. In the present study, the Qinling mountains and Dabie Mountains separate Zaoyang city of Hubei from the other 6 sites. There was great genetic differentiation between Hubei and the other populations, which might result from a limited dispersal of aphids. Similar phenomena of genetic isolation resulting from by geographical barriers were also found in *Eriosoma lanigerum* [70], two tansy-feeding aphids (*Macrosiphoniella tanacetaria* and *Metopeurum fuscoviride*) [33], and *Diuraphis noxia* [34].

Populations of *S. avenae* in China might be classified into 4 clusters based on our research results. The population from Shanxi had less communication with other populations because of Taihang mountain, which may have obstructed the migration of the wheat aphids. However, the geographic location of Shanxi is adjacent to Henan, Hebei, and Shandong, and the location of sample collection for Shanxi was Yuncheng, which is at the southern limit of the Taihang mountain range, so gene flow was affected relatively little and there was overlap between Shanxi, Henan, and Shandong in the FCA analysis (Figure 3).

## Conclusions

In the present study, we analyzed the population genetics and predicted the gene flow among the seven different *S. avenae* geographical populations in China using microsatellite marker technology. The genetic diversity of the Henan and Shandong populations was higher than the others populations. The genic diversity of the Hebei and Beijing populations were the lowest. For genetic differentiation, there was high or significant differentiation among the Hubei population and the others. The genetic differentiation between Beijing and Hebei, and Henan and Shandong were both at very low levels, indicating that the gene flow occurs frequently between these populations. Finally, the 7 *S. avenae* geographic populations can be divided into 4 clusters: (i) Hubei, (ii) Shanxi, (iii) Beijing and Hebei, and (iv) Shandong, Henan, and Jiangsu. The present results provide a basis for efforts to optimize Integrated Pest Management programs through adapting control methods which target the biological traits shared by various populations from the same genotype.

## Supporting Information

File S1 Microsatellite genotypes of the seven *Sitobion avenae* populations in China. “individual ID”, the tag of each tested aphid individual. “sampling location”, the corresponding location where each aphid individual was collected. The other ten columns were the corresponding allele sizes of each aphid individual at each microsatellite loci.
(XLSX)
